# Supplementary material for: Diverse Partners of the Partitioning ParB Protein in Pseudomonas aeruginosa
Source: Microbiol Spectr. 2023 Jan 9;11(1):e04289-22. doi: 10.1128/spectrum.04289-22 (PMC9927451; doi:10.1128/spectrum.04289-22)
Supplement: Supplemental file 4 — Supplemental material. Download spectrum.04289-22-s0004.pdf, PDF file, 2.6 MB [file spectrum.04289-22-s0004.pdf]

SUPPLEMENTARY MATERIAL

Diverse partners of the partitioning ParB protein in *Pseudomonas aeruginosa*

Adam Kawalek, Krzysztof Glabski, Aneta Agnieszka Bartosik, Dominika Wozniak, Magdalena Kusiak, Jan Gawor, Karolina Zuchniewicz, Grazyna Jagura-Burdzy

SUPPLEMENTARY MATERIALS AND METHODS

Construction of *P. aeruginosa* deletion mutants by homologous recombination

The pAKE600 suicide vector (1) was used to prepare alleles of genes of interest (Table S7). Derivatives were transformed into *E. coli* S17-1 strain. The pAKE600 carries $oriT_{RK2}$ and may be mobilized into *P. aeruginosa* by RK2 conjugational system inserted into the S17-1 chromosome. The pAKE600, based on the ori_{MB1}, is unable to replicate in *P. aeruginosa*, so it can only integrate into chromosome using the region of homology, to confer carbenicillin resistance to the cells. The second crossing-over event may lead to the excision of the integrated plasmid and allele exchange. The overnight cultures of *E. coli* S17-1 strain with pAKE600 derivatives (donor) and *P. aeruginosa* PAO1161 Rif^R (or derivatives) recipient cells were mixed in proportions 1:1 or 2:1 (donor:recipient) and 100 µl of such mixture was transferred on L-agar plate. After overnight incubation at 37°C the bacterial lawn was suspended in 1 ml of sterile L-broth, the suspension was diluted and plated on the L-agar plates with selection (rifampicin and carbenicillin). Integrants were re-streaked on the same selective plate and the integration was verified using colony PCR. The integrants were used to inoculate L-broth with 10% sucrose (w/v), incubated overnight at 28°C to induce excision of pAKE600 carrying *sacB* toxic for the cells under such conditions. Cells were plated on L-agar with 10% sucrose (w/v) and carbenicillin-sensitive strains, grown on such plates, were subjected to PCR to screen for colonies with exchanged allele. Primers used for strain verification were designed to anneal to the regions outside the sequences used as the regions of homology.

Fluorescence microscopy and image analysis

P. aeruginosa strains expressing fluorescent protein fusions were cultured at 37°C in 100 ml flasks sealed with a cotton plug in 20 ml of LB or M9 medium with the appropriate additives, shaken at 200 rpm. Overnight cultures were diluted 1:200 in a fresh medium. When the OD_{600} reached 0.3-0.5, 0.5 ml of the cultures were fixed by addition of 0.5 ml of 2.8% paraformaldehyde, 0.04% glutaraldehyde in PBS (137mM NaCl, 2.7mM KCl, 10mM Na_2HPO_4 , 1.8mM K_2HPO_4). The cells were incubated at 4°C for at least 30 min (to overnight), centrifuged (5 min, 2500g) and the pellet was washed with 1x PBS. The pellet was finally resuspended in approximately 10 µl of PBS. The cell suspension was applied to a polylysine coated glass slide and covered with a coverslip. Cells were analyzed using a Zeiss Imager M2 microscope, a 100x 1.30 NA Plan-Neofluar objective, a Zeiss AxioCam MRc5 camera and AxioVision (AxioVs40 V 4.8.2.0, Carl Zeiss MicroImaging) software. CFP signal was visualized with a 436/20 nm bandpass excitation filter, a 455 nm dichroic mirror, and a 480/40-nm bandpass emission filter. SYTO9 and GFP signals were visualized with a 470/40 nm bandpass excitation filter, a 495 nm dichroic mirror, and a 525/50-nm bandpass emission filter. The YFP signal was visualized using a 500/20 nm excitation filter, a 515 nm dichroic mirror and a 535/30 nm bandpass emission filter. The propidium iodide signal was imaged using a 572/25 nm excitation filter, a 590 nm dichroic mirror and a 629/62 nm bandpass emission filter. For YFP-ParB and $ParB_{P1}$-GFP, a series of images (Z-stacks) were acquired (3-5 images, 0.25 µm distance) and merged (Z-projection) using maximum intensity mode.

The images were analyzed using ImageJ 1.51e (https://imagej.nih.gov/ij/). Quantitative fluorescence analysis in individual cells was performed using MicrobeJ 5.13m (https://www.microbej.com/) (2). Before preparation of hyperstacks (a combination of a bright-field and fluorescence image), the contrast of bright-field images was enhanced using the following macro:

```
run("16-bit");
run("Subtract Background...", "rolling=10 light");
run("Smooth");
run("Brightness/Contrast...");
run("Enhance Contrast", "saturated=0.35");
run("Apply LUT");
```

In bright field images, cell contours were identified using the fit shape option for rod-shaped bacteria with the following parameters: area 0.75-4.5 µm^2; length > 0.5 µm; width (symmetry) > 0.4 µm; circularity > 0; curvature 0-0.2; sinuosity > 0, angularity 0-0.2; solidity > 0.92. Foci were subsequently detected in the areas of the images limited by the outline of the bacteria in the images containing fluorescence signal using point mode, with a detection tolerance of 10 and foci intensity > 20. However these two last parameters had to be adjusted for each fusion with fluorescent protein and even for each batch of the images. The number of foci and their position along the long axis of the cell were counted for each cell. For the orientation of the cells, the first pole was assumed to be in the half of the cell with the higher fluorescence signal. The distribution of fluorescence in cells was presented in the form of fluorescence profiles (0.2 µm stripes along the long axis of the cell, sorted by cell length). The results of MicrobeJ analyses were exported and modified using Inkscape (https://inkscape.org/). For analysis of *ori* region segregation using orthogonal $ParB_{P1}$-$parS_{P1}$ system relation between the proportion of cells with two $ParB_{P1}$-GFP foci and cell size was determined using a custom R script.

Analysis of anucleate cell content

Cultures were grown until OD_{600} reached 0.3-0.5 in the indicated medium. Cells were fixed in 5% formaldehyde for 5 min at room temperature (0.5 ml of culture added to 78 µl 37% formaldehyde), pelleted by centrifugation (5 min, 2500x g), resuspended in 200 µl of L broth and 200 µl of 96% ethanol was added. Cells were mixed by inversion, incubated 5 min at room temperature, pelleted (5 min, 2500x g), resuspended in 450 µl of PBS containing 20 µl/ml RNAse A (DNAse free) and kept at 4ºC for 16h. Subsequently, 0.15 µl of 1.67 mM SYTO 9/ 18.3 mM propidium iodide mix (Live-Dead BacLight Bacterial Viability Kit, Thermo Scientific, ref. L7007) was added, samples were mixed and left for 5 min at room temperature. Cells were concentrated (5 min, 2500x g), and subjected to a microscopy analysis. While SYTO 9 green gave a faint signal with fixed cells, the staining resulted in a very bright signal with live bacteria, which allowed to exclude non-fixed (e.g. contaminating) bacteria in samples. The number of anucleate cells (cells which didn't stain with propidium iodide) was counted manually on each image. The total number of cells on images was counted on bright-field images using the ImageJ macro:

```
run("Subtract Background...", "rolling=20 light");
run("8-bit");
setAutoThreshold("Default");
setOption("BlackBackground", false);
run("Convert to Mask");
run("Watershed");
run("Analyze Particles...", "size=50-Infinity pixel show=Outlines exclude clear summarize");
```

In our hands the difference between the number of cells counted using the macro and counted manually in several images was always lower than 10%. During the image acquisition care was taken to avoid cell clumps.

SUPPLEMENTARY FIGURES:

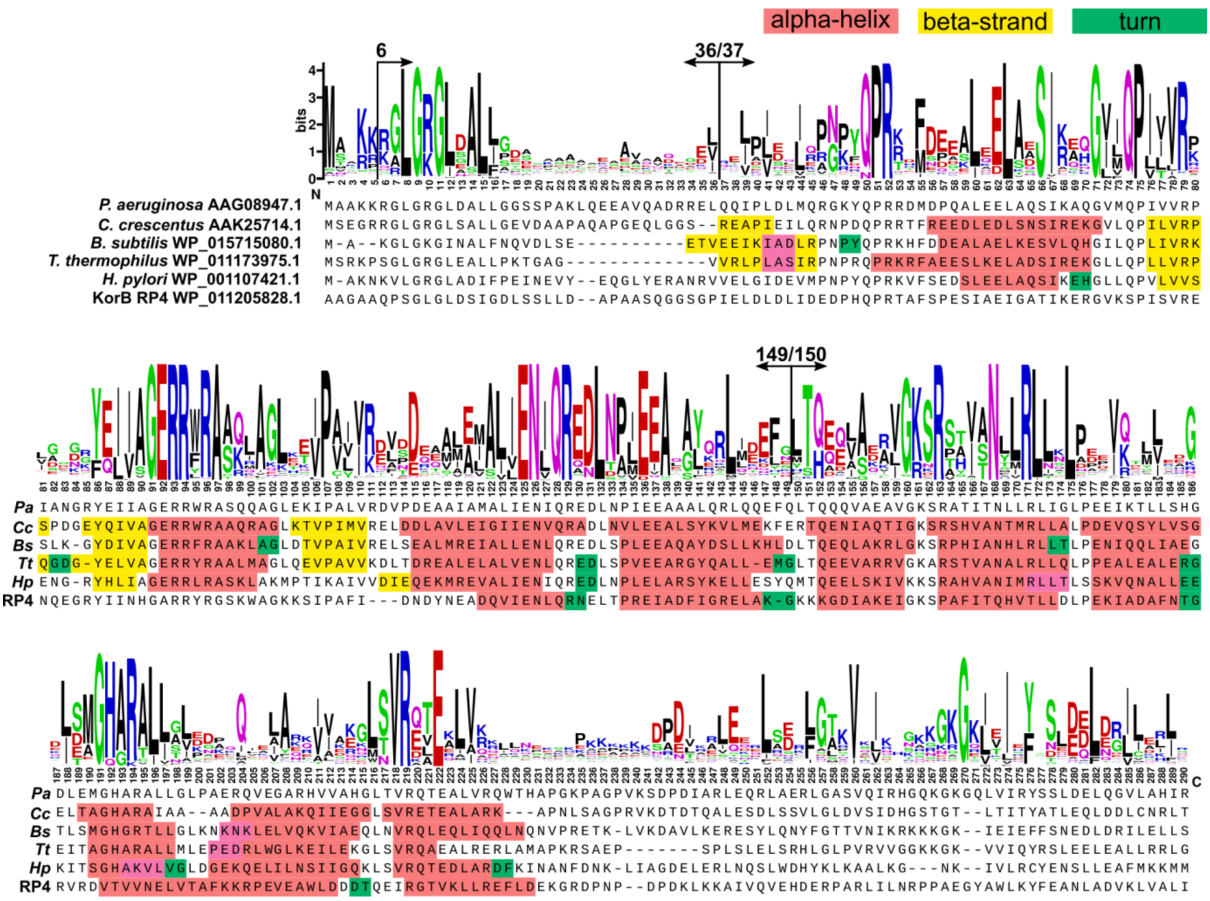

Figure S1 Sequence logo, representing the conservation of *P. aeruginosa* ParB amino acids. Clustered genes encoding proteins with similarity to *P. aeruginosa* ParA and ParB were identified using Multigeneblast (3) in representative and reference bacterial genomes (Refseq database, release 91, total of 1748 genomes) yielding 821 genomes encoding orthologous proteins. MultiGeneBlast results were converted using MGBparser.py (https://github.com/jrjhealey/MultiGeneBlastParser) and a custom R script was used to extract ParB's accession numbers. Sequences of ParB's were subsequently aligned with *P. aeruginosa* ParB using MAFFT v7.475 (2020/Nov/23) (4) with the use of '--keeplength' and '--add' options. Logo was subsequently generated using Weblogo (https://weblogo.berkeley.edu/logo.cgi). Alignment below represents comparison of *P. aeruginosa* ParB sequence with those for which the structure was solved: *Caulobacter crescentus* (PDB ID: 6T1F, (5)), *Bacillus subtilis* (6SDK, (6)), *Thermus thermophilus* (1VZ0, (7)), *Helicobacter pylori* (4UMK, (8)) as well as KorB protein of the broad-host-range plasmid RP4 (1R71, (9)). Secondary structure elements were highlighted using the indicated colors.

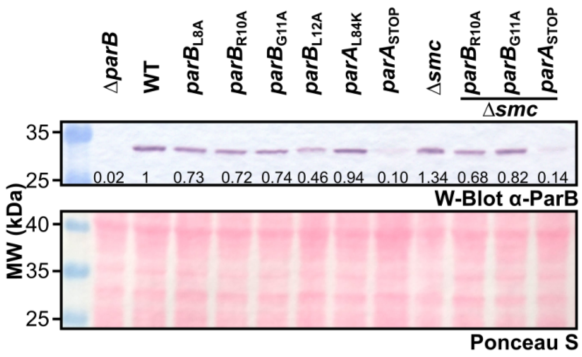

Figure S2 ParB protein levels in the cells of indicated strains. ParB content in cell extracts from exponentially growing cultures was analyzed by Western blotting with polyclonal anti-ParB antibodies. Ponceau S-stained membrane is shown as a loading control. Numbers indicate the relative ParB abundance in strains based on signal quantification on the blots using ImageJ, normalized with the signal of the Ponceau S staining. Experiment was repeated 3 times with the same trends.

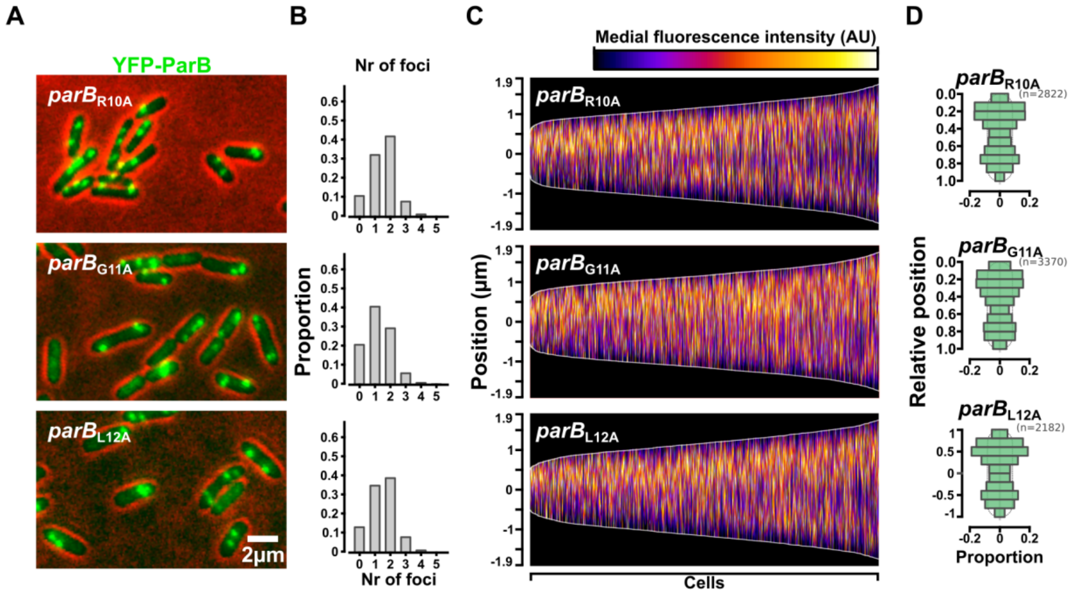

Figure S3 Addendum to Figure 2A-D. Microscopic analysis of ParB localization in *P. aeruginosa* mutant cells. Bacteria were grown without IPTG in LB medium at 37ºC till logarithmic phase of growth (OD_{600} of 0.4). (**A**) Representative microscope images showing cellular localization of YFP-ParB mutants analyzed in strains carrying the same mutations in the chromosome. (**B**) Distribution of the number of YFP-ParB foci per cell in analyzed strains. Foci were detected in fluorescence images using MicrobeJ with the same criteria for all strains. The differences between strains and WT are significant (Chi-squared goodness of fit test). (**C**) Distribution of YFP-ParB fluorescence along the long axis of the cells. Profiles of YFP-ParB fluorescence of individual cells, sorted based on cell length, and oriented in a way in which the top (first pole) represents the cell halve with higher mean fluorescence signal. (**D**) Relative position of YFP-ParB foci analyzed in two foci containing cells. Each cell was split in ten bins, along the long axis and the histogram was mirrored.

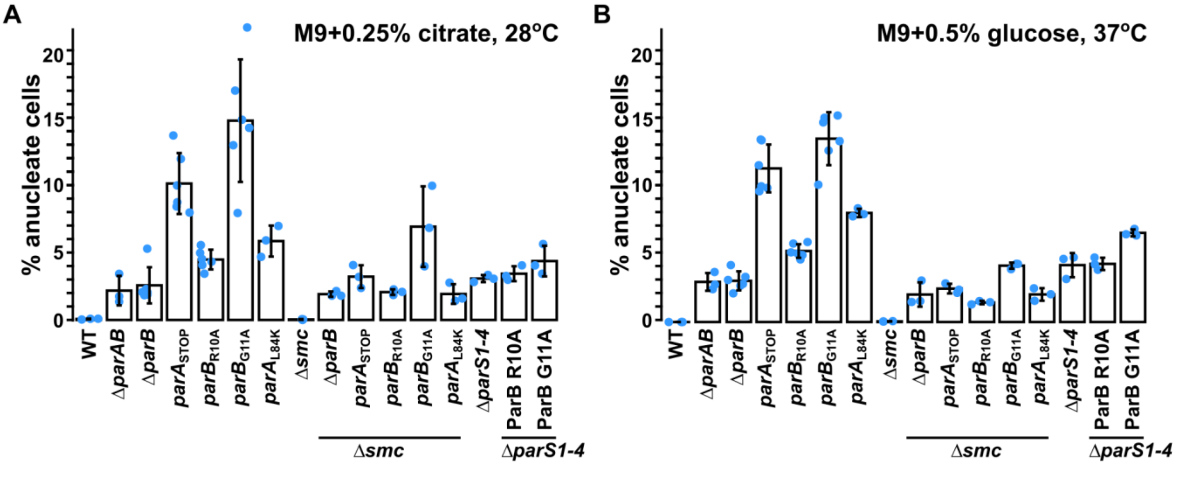

Figure S4 The detrimental effect of unseparated ParB complexes on the cells doesn't depend on the growth conditions. Percentage of anucleate cells in cultures of indicated strains growing in M9 medium containing (**A**) 0.25% sodium citrate at 28ºC, (**B**) 0.5% glucose at 37ºC. Cells were collected at the exponential phase of growth (OD_{600} ~0.3), fixed, stained with propidium iodide and analyzed by fluorescence microscopy. Data represent mean ±SD and blue dots represent the individual measurements. At least 2500 cells were analyzed for each culture.

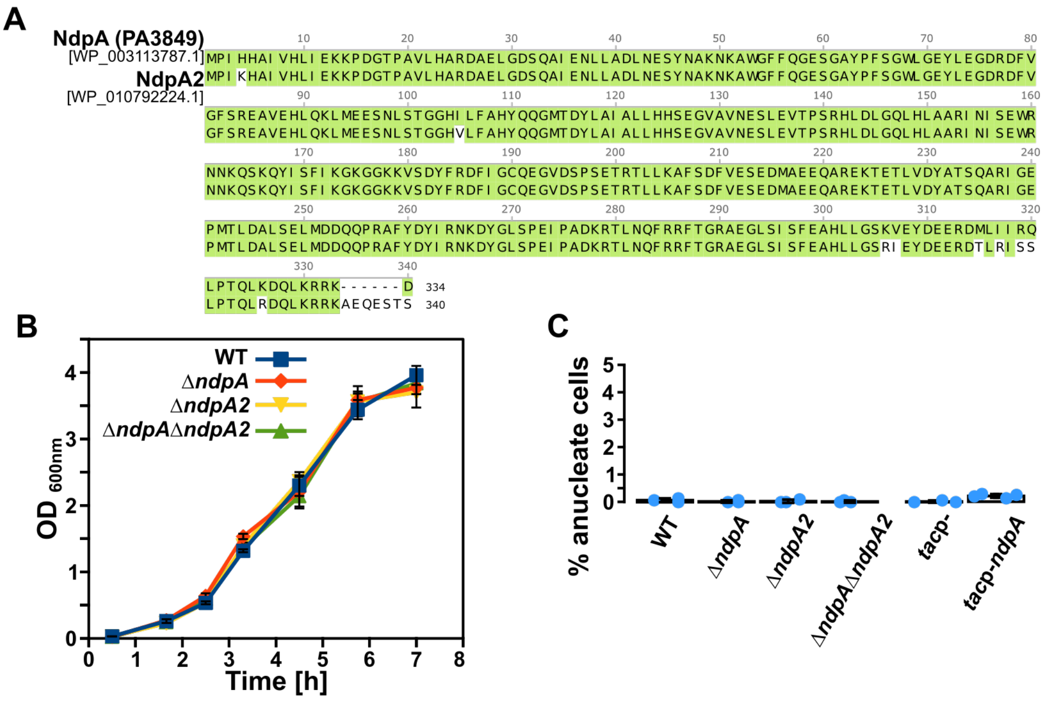

Figure S5 Lack of NdpA proteins does not have a major effect on growth and chromosome segregation in *P. aeruginosa*. (**A**) Alignment of *P. aeruginosa* PAO1161 NdpA and NdpA2. NdpA2 is encoded on the PAPI-1 family integrative conjugative element ICE*Pae*1161 (10). (**B**) Growth of the indicated *P. aeruginosa* strains in LB medium. Data represents mean OD_{600} ±SD for 3 replicates. (**C**) Percentage of anucleate cells in exponentially growing cultures (OD_{600} ~0.5) of indicated strains assessed by microscopic analysis of the DNA presence after propidium iodide staining. 0.2 mM IPTG was used for induction of *tac* promoter in case of strains with vectors. Data represent mean ±SD and blue dots represent the individual measurements. At least 3000 cells were analyzed for each culture.

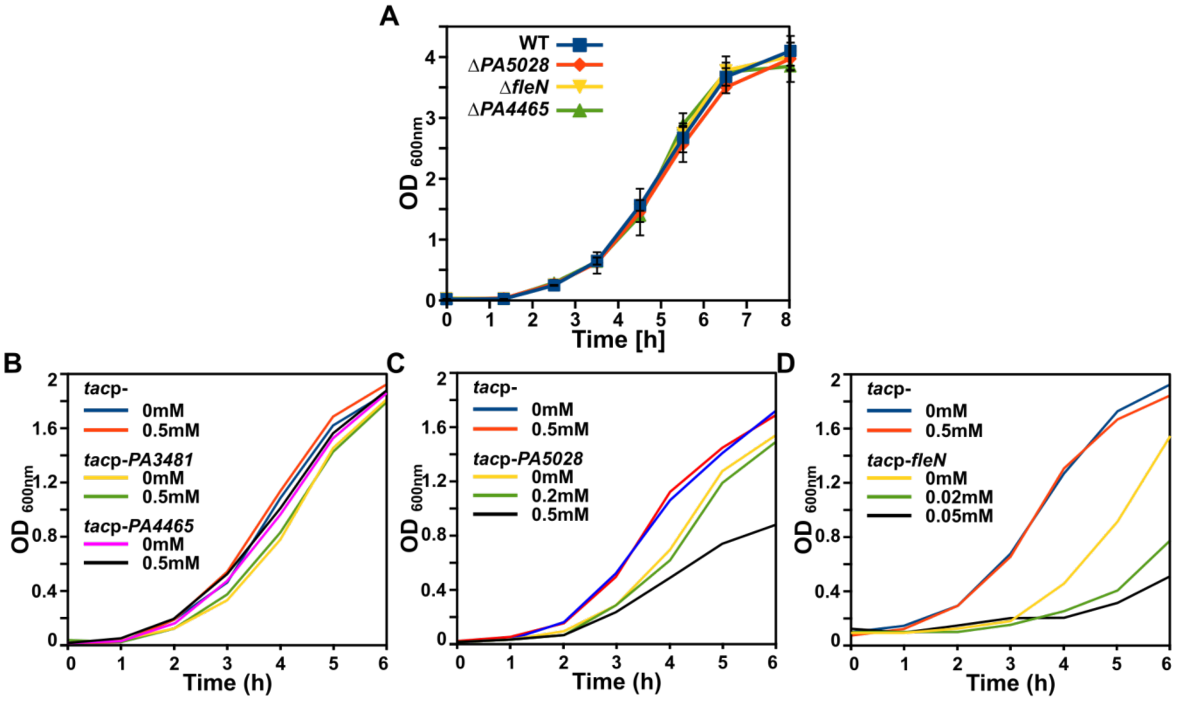

Figure S6 Effect of gene deficiency or overexpression on growth of *P. aeruginosa* cells. (**A**) Growth of indicated deletion strains in LB medium at 37°C. Data represent mean optical density at 600 nm ±SD from 3 cultures. (**B**) Effect of gene overexpression from *tac* promoter in medium with different concentrations of inducer (IPTG). Data represent mean optical density at 600 nm from 3 cultures.

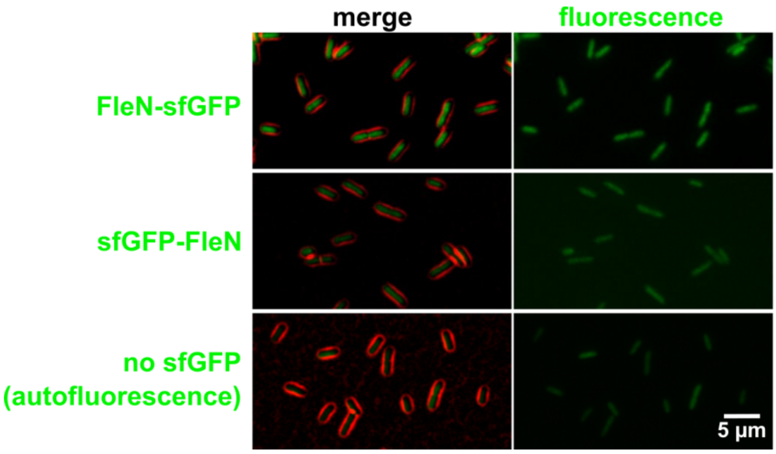

Figure S7 Fluorescence microscopy analysis of FleN localization in *P. aeruginosa*. Cells expressing *fleN-sfGFP*, *sfGFP-fleN* under control of *tac*p as well as control strain with empty vector (pAMB9.37 *tac*p-) were grown in medium without inducer (IPTG) and imaged.

TABLES AND TABLE LEGENDS:

Table S1 Identification of ParB interacting proteins using co-immunoprecipitation and mass-spectrometry analysis. *P. aeruginosa* cells producing FLAG-ParB and WT (control) strains were used together with anti FLAG antibodies. Recovered proteins were identified using mass spectrometry. Data represent the number of peptides and Mascot scores for individual samples. Proteins with a Mascot score of 100 in at least one of the samples were kept. BACTH vectors used for particular proteins to analyze the interactions with ParB are listed in Table S7. (.xlsx file)

Table S2 Identification of ParB interacting proteins using a screening of BACTH library. BACTH vectors used for particular proteins to analyze the interactions with ParB are listed in Table S7. (.xlsx file)

Table S3 ParB ChIP-seq peaks outside the *parS1-parS4* region identified for WT *P. aeruginosa* PAO1161 strain in this study. Peaks were identified using MACS2 and filtered to only include those with fold enrichment >3. Peaks around *parS1-4* (-25 kbp to +140 kbp) region were excluded. (.xlsx file).

Table S4 Bacterial strains used in this study

Strain name	Description / Relevant genotype	Origin
Escherichia coli		
DH5α	F^- Φ80*lacZ*ΔM15 Δ(*lacZYA-argF*) *U169 recA1 endA1 hsdR17* (r_{K-}, m_{K+}) *phoA supE44 λ thi-1 gyrA96 relA*	(11)
S17-1	*pro* Δ*hsdR hsdM*$^+$ *recA* Tp^R Sm^R ΩRP4-Tc::Mu Kn::Tn7	(12)
BTH101	F^- *cya-99 araD139 galE15 galK16 rpsL1 (Str^r) hsdR2 mcrA1 mcrB1*	(13)
BL21 (DE3)	F^- *ompT hsdS*B ($r_B^-m_B^-$) *gal dcm* (λDE3)	Novagen
Pseudomonas aeruginosa		
PAO1161 (WT)	Rif^R, *leu-,* PAO1 derivative	(10)
parA$_{STOP}$	PAO1161 derivative, *parA* gene modified to contain two STOP codons directly downstream of ORF start codon, no ParA synthesis	(14)
Δ*parS1-4*	PAO1161 with *parS1*, *parS2*, *parS3*, *parS4*, modified to block ParB binding; named *parS*mut15 in the original paper	(15)
parA$_{L84K}$	PAO1161 expressing ParA L84K variant, disturbed in self-interaction and interactions with ParB	(14)

Table S5 *Pseudomonas aeruginosa* PAO1161 derivatives constructed in this study

Strain name/genotype	Plasmid(s) used for allele exchange, description
Δ*parB*	pAKW0004, *parB* (*PA5562*) deletion
Δ*parAB*	pAAB0813, *parAB* (*PA5563-PA5562*) deletion
$parB_{L8A}$	pAKW1105, produces ParB L8A variant
$parB_{R10A}$	pAKW1107, produces ParB R10A variant
$parB_{G11A}$	pAKW1108, produces ParB G11 variant
$parB_{L12A}$	pAKW1109, produces ParB L12A variant
Δ*parS1-4* $parA_{L84K}$	Δ*parS1-4* derivative, pAAB0298, produces ParA L84K variant
Δ*parS1-4* $parB_{L8A}$	Δ*parS1-4* derivative, pAKW1105, produces ParB L8A variant
Δ*parS1-4* $parB_{R10A}$	Δ*parS1-4* derivative, pAKW1107, produces ParB R10A variant
Δ*parS1-4* $parB_{G11A}$	Δ*parS1-4* derivative, pAKW1108, produces ParB G11 variant
Δ*parS1-4* $parB_{L12A}$	Δ*parS1-4* derivative, pAKW1109, produces ParB L12A variant
Δ*smc*	pMKU0316, *smc* (*PA1527*) deletion
Δ*smc* $parA_{STOP}$	pMKU0316, pAAB0331, *smc* (*PA1527*) deletion, no ParA synthesis
Δ*smc* Δ*parB*	pMKU0316, pAKW0004, *smc* (*PA1527*) and *parB* (*PA5562*) deletions
Δ*smc* $parA_{L84K}$	pMKU0316, pAAB0298, *smc* (*PA1527*) deletion, produces ParA L84K variant
Δ*smc* $parB_{L8A}$	pMKU0316, pAKW1105, *smc* (*PA1527*) deletion, produces ParB L8A variant
Δ*smc* $parB_{R10A}$	pMKU0316, pAKW1107, *smc* (*PA1527*) deletion, produces ParB R10A variant
Δ*smc* $parB_{G11A}$	pMKU0316, pAKW1108, *smc* (*PA1527*) deletion, produces ParB G11 variant
Δ*smc* $parB_{L12A}$	pMKU0316, pAKW1109, *smc* (*PA1527*) deletion, produces ParB L12A variant
flag-parB	pAKW0002, produces FLAG-ParB, used for identification of ParB partners using co-immunoprecipitation
Δ*minC*	pKGB0275, *minC* (*PA3243*) deletion
Δ*minD*	pKGB0167, *minD* (*PA3244*) deletion
Δ*minCDE*	pAKW0896, *minCDE* (*PA3243-PA3245*) operon deletion
Δ*fleN*	pKGB0179, *fleN* (*PA1454*) deletion
Δ*PA5028*	pKGB0236, *PA5028* deletion
Δ*PA4465*	pAAB0755, *PA4465* deletion, Tet^R
Δ*fleN* Δ*PA5028* Δ*PA4465*	pKGB0179, pKGB0236, pAAB0755, triple deletion strain, Tet^R
Δ*mksBEF*	pAKW1207, *mksBEF* (*PA4684-PA4686*) operon deletion
Δ*mksBEF* Δ*fleN*	pKGB0179, pAKW1207, deletion of *mksBEF* (*PA4684-PA4686*) and *fleN*
Δ*mksBEF* Δ*PA5028*	pKGB0236, pAKW1207, deletion of *mksBEF* (*PA4684-PA4686*) and *PA5028*
Δ*mksBEF* Δ*PA4465*	pAAB0755, pAKW1207, deletion of *mksBEF* (*PA4684-PA4686*) and *PA4465*, Tet^R
Δ*mksBEF* Δ*fleN* Δ*PA5028* Δ*PA4465*	pKGB0179, pAKW1207, pKGB0236, pAAB0755, deletion of *mksBEF* (*PA4684-PA4686*), *fleN*, *PA5028, PA4465*, Tet^R
Δ*parAB* Δ*fleN*	pAAB0813, pKGB0179, deletion of *parAB* (*PA5563-PA5562*) and *fleN*
Δ*parAB* Δ*PA5028*	pAAB0813, pKGB0236, deletion of *parAB* (*PA5563-PA5562*) and *PA5028*
Δ*parAB* Δ*PA4465*	pAAB0813, pAAB0755, deletion of *parAB* (*PA5563-PA5562*) and *PA4465*, Tet^R
*araBAD*p-*sfGFP*	pAKW0861, *araC-araBAD*p-*sfGFP* inserted between PA5412/PA5413,
Δ*ndpA*	pKGB0474, *ndpA* (*PA3849*) deletion
Δ*ndpA2*	pAKW0796, *ndpA2* (*D3C65_RS24150*) deletion
Δ*ndpA* Δ*ndpA2*	pKGB0474, pAKW0796, *ndpA* (*PA3849*) and *ndpA2* (*D3C65_RS24150*) deletion
PA0069-$parS_{P1}$(82R)	pAKW1217, $parS_{P1}$ between *PA0069* and *PA0070*
Δ*smc PA0069*-$parS_{P1}$(82R)	pMKU0316 pAKW1217, *smc* (*PA1527*) deletion, $parS_{P1}$ between *PA0069* and *PA0070*
$parB_{G11A}$ *PA0069*-$parS_{P1}$(82R)	pAKW1108, pAKW1217, produces ParB G11 variant, $parS_{P1}$ between *PA0069* and *PA0070*
Δ*smc* $parB_{G11A}$ *PA0069*-$parS_{P1}$(82R)	pMKU0316, pAKW1108, pAKW1217, *smc* (*PA1527*) deletion, produces ParB G11 variant, $parS_{P1}$ between *PA0069* and *PA0070*

Table S6 Plasmids used in this study

Plasmid name	Description and relevant features	Origin
pAKE600	suicide vector used for allele exchange in *Pae*; *oriV*$_{MB1}$, *oriT*$_{RK2}$ *sacB* casette, Amp^R	(1)
pAAB0331	pAKE600 with *parA*$_{STOP}$ allele (no ParA synthesis)	(16)
pAAB0298	pAKE600 with *parA*$_{L84K}$ allele	(14)
pKAB241	*araC-araBAD*p-*flag-parB*, *oriV*$_{MB1}$, Amp^R	(17)
pKRP12	*oriV*$_{MB1}$, contains Tet^R cassette, Tet^R, Amp^R	(18)
pKAB601	pAKE600 with *PA5412*/*PA5413* intergenic region of PAO1161 with *araC*-*araBAD*p-*mcs*-*rrnB*t cassette inserted into PstI site, Amp^R	(17)
pUC19	cloning vector, *ori*$_{MB1}$, Amp^R	(19)
pBGS18	cloning vector, *ori*$_{MB1}$, Kan^R	(20)
pGBT30	expression vector, *lacI*^{q}-*tac*p-*mcs*, *ori*$_{MB1}$, Amp^R,	(21)
pOMB3.104	pUC18 with *lacO-parSP1-lacO*, *parS*$_{P1}$ cloned as EcoRI fragment from pBEF143 (22)	Monika Mitura
pOKB3	*trp*p-*parB*$_{P1}$-*GFP*, pBEF104 (23) derivative, *ori*$_{ColE1}$, Amp^R	Juszczuk Olga
pKAB8	expression vector with *araC-araBAD*p, *ori*$_{IncA/C}$, Chl^R	(24)
pAMB9.37	pBBR1MCS-1 (21) derivative with inserted *lacI*^{q} *tacp*, *ori*$_{IncA/C}$, Chl^R	(26)
pABB1.2	pAMB9.37 with *tacp-parB* transcriptional fusion	(14)
pET28a	expression vector *T7*p, *lacO*, *his*$_6$-tag, T7tag, *ori*$_{MB1}$, Kan^R	Clontech
pEYFP	*tac*p-*yfp*, ori$_{MB1}$, Amp^R	Clontech
pECFP	*tac*p-*yfp*, ori$_{MB1}$, Amp^R	Clontech
pUT18C	*lac*p–*cyaT18–mcs*, *ori*$_{ColE1}$, Amp^R	(13)
pUT18	*lac*p–*mcs–cyaT18*, *ori*$_{ColE1}$, Amp^R	(13)
pKT25	*lac*p–*cyaT25–mcs*, *ori*$_{p15}$, Kan^R	(13)
pKNT25	*lac*p–*mcs–cyaT25*, *ori*$_{p15}$, Kan^R	(13)
pLKB4	pUT18C(13) with modified *mcs*, *lacp-cyaT18-mcs*	(27)
pKGB4	pUT18 (13) with modified *mcs*, *lacp-mcs-cyaT18*	(14)
pLKB2	pKT25 (13) with modified *mcs*, *lac*p-*cyaT25-mcs*	(27)
pKGB5	pKNT25(13) with modified *mcs*, *lac*p-*mcs-cyaT25*	(14)
pMKB5.1	pLKB4 with *cyaT18–parA*	(14)
pMKB5.3	pUT18 with *parA–cyaT18*	(14)
pMKB6.1	pKNT25 with *parA–cyaT25*	(14)
pLKB220	pLKB2 with *cyaT25–parA*	(14)
pMKB5.2	pLKB4 with *cyaT18–parB* (used for experiments on Fig. 4)	(14)
pMKB5.4	pUT18 with *parB–cyaT18*	(14)
pLKB233	pLKB2 with *cyaT25–parB*	(14)
pMKB6.2	pKNT25 with *parB–cyaT25*	(14)
pKT25-zip	*lac*p-*cyaT25-GCN4* (leucine zipper), *ori*$_{p15}$, Kan^{R,}	(13)
pUT18C-zip	*lac*p -*cyaT18-GCN4* (leucine zipper), *ori*$_{ColE1}$, Amp^R,	(13)
pKAB18.1	pLKB4 with *cyaT18–mexZ* (*PA2020*)	(24)
pKAB25.1	pLKB2 with *cyaT25–mexZ* (*PA2020*)	(24)
BBa_I746908	pSB1C3 (*ori*$_{MB1}$, Chl^R) with *araC-araBAD*p-*rbs*$_{Ba\_B0034}$-*sfgfp*	iGEM collection

Table S7 Plasmids constructed in this study. PCR was performed using PAO1161Rif^{R} genomic DNA as a template, unless stated otherwise.

Name	Relevant features and construction
**pAKE600 derivatives (used for *Pae* genome modifications)**	
pAKW0004	*parB* (*PA5562*) deletion cassette; HindIII-PstI digested PCR product (primers #1 and #2) and PstI-SalI digested PCR product (primers #3 and #4) inserted simultaneously between HindIII and SalI sites in pAKE600.
pAAB0813	*parBA* (*PA5563-PA5562*) deletion cassette; EcoRI/PstI digested PCR product (primers #5 and #6) replaced EcoRI/PstI fragment in pAKW0004.
pAKW1105	*parB*$_{L8A}$ mutagenesis cassette; PCR fragments amplified with primers #1/ #8 and #7/ #19 were mixed in an overlap PCR (primers #1 and #19). HindIII and SalI digested product was inserted between HindIII and SalI sites in pAKE600. Introduction of a Eco52I site through silent mutagenesis, facilitated the identification of clones with this allele.
pAKW1107	*parB*$_{R10A}$ mutagenesis cassette; PCR fragments amplified with primers #1/ #12 and #11/ #19 were mixed in an overlap PCR (primers #1 and #19). HindIII and SalI digested product was inserted between HindIII and SalI sites in pAKE600. Introduction of a XbaI site through silent mutagenesis, facilitated the identification of clones with this allele.
pAKW1108	*parB*$_{G11A}$ mutagenesis cassette; PCR fragments amplified with primers #1/ #14 and #13/ #19 were mixed in an overlap PCR (primers #1 and #19). HindIII and SalI digested product was inserted between HindIII and SalI sites in pAKE600. Introduction of a BssHII site through silent mutagenesis, facilitated the identification of clones with this allele.
pAKW1109	*parB*$_{L12A}$ mutagenesis cassette; PCR fragments amplified with primers #1/ #16 and #15/ #19 were mixed in an overlap PCR (primers #1 and #19). HindIII and SalI digested product was inserted between HindIII and SalI sites in pAKE600. Introduction of a NsiI site through silent mutagenesis, facilitated the identification of clones with this allele.
pMKU0316	*smc* (*PA1527*) deletion cassette; EcoRI/HindIII digested PCR product (primers #20 and #21) and HindIII/SalI digested PCR product (primers #22 and #23) inserted simultaneously between EcoRI and SalI sites in pAKE600.
pAKW0002	*flag-parB* mutagenesis cassette; PCR fragments amplified with primers #1/ #17 and #18/ #19 (pKAB241) were mixed in an overlap PCR (primers #1 and #19). HindIII and SalI digested product was inserted between HindIII and SalI sites in pAKE600.
pKGB0275	*minC* (*PA3243*) deletion cassette; EcoRI/HindIII digested PCR product (primers #24 and #25) and HindIII/BamHII digested PCR product (primers #26 and #27) inserted simultaneously between EcoRI and SalI sites in pAKE600.
pKGB0167	*minD* (*PA3244*) deletion cassette; HindIII/NcoI digested PCR product (primers #28 and #29) and NcoI/BamHII digested PCR product (primers #30 and #31) inserted simultaneously between HindIII and BamHI sites in pAKE600.
pAKW0896	*minCDE* (*PA3243-PA3245*) deletion cassette; HindIII/BamHI digested PCR product (primers #32 and #33) replaced HindIII/BamHI fragment in pKGB0275.
pKGB0179	*fleN* (*PA1454*) deletion cassette; EcoRI/HindIII digested PCR product (primers #34 and #35) and HindIII//BamHII digested PCR product (primers #36 and #37) inserted simultaneously between EcoRI and BamHI sites in pAKE600.
pKGB0236	*PA5028* deletion cassette; EcoRI/HindIII digested PCR product (primers #38 and #39) and HindIII//BamHII digested PCR product (primers #40 and #41) inserted simultaneously between EcoRI and BamHI sites in pAKE600.
pAAB0755	*PA4465* deletion cassette (with Tet^{R}); EcoRI/HindIII digested PCR product (primers #42 and #43) and HindIII//BamHII digested PCR product (primers #44 and #45) inserted simultaneously between EcoRI and BamHI sites in pAKE600. Subsequently Tet^{R} cassette, excised from pKRP12 was inserted into the HindIII site.
pAKW1207	*mksBEF* (*PA4684-PA4686*) deletion cassette; EcoRI/HindIII digested PCR product (primers #46 and #47) and HindIII//BamHII digested PCR product (primers #48 and #49) inserted simultaneously between EcoRI and BamHI sites in pAKE600.
pAKW0861	allows insertion of *araC-araBAD*p-*sfGFP* between *PA5412*/*PA5413*, pKAB601 derivative.
pKGB0474	*ndpA* (*PA3849*) deletion cassette; EcoRI/HindIII digested PCR product (primers #50 and #51) and HindIII//BamHII digested PCR product (primers #52 and #53) inserted simultaneously between EcoRI and BamHI sites in pAKE600.
pAKW0796	*ndpA2* (*D3C65_RS24150*) in PAO1161 genome) deletion cassette; EcoRI/HindIII digested PCR product (primers #54 and #55) and HindIII//BamHII digested PCR product (primers #56 and #57) inserted simultaneously between EcoRI and BamHI sites in pAKE600.
pAKW1217	insertion of *parS*$_{P1}$ between *PA0069* and *PA0070*; EcoRI/HindIII digested PCR product (primers #58 and #59) and HindIII//BamHII digested PCR product (primers #60 and #61) inserted simultaneously between EcoRI-BamHI sites in pAKE600 to yield plasmid pAKW1203. *parS1*$_{P1}$ amplified on pOMB3.104 using primers #62 and #63 was subcloned as a SmaI fragment into pUC19 to yield pAKW1204. *parS1*$_{P1}$ excised from pAKW1204 using NsiI, inserted into the PstI site of pAKW1203.
**BACTH vectors (pUT18C derivatives)**	
pAKW1075	*cyaT18-parB*; PCR product amplified using primers #66 and #67 inserted between EcoRI-BamHI sites in pLKB4
pAKW1071	*cyaT18-parB*$_{238-290}$; PCR product amplified using primers #70 and #67 inserted between EcoRI-BamHI sites in pLKB4
pAKW1070	*cyaT18-parB*$_{150-290}$; PCR product amplified using primers #69 and #67 inserted between EcoRI-BamHI sites in pLKB4
pAKW1080	*cyaT18-parB*$_{37-290}$; PCR product amplified using primers #68 and #67 inserted between EcoRI-BamHI sites in pLKB4
pAKW1069	*cyaT18-parB*$_{6-290}$; PCR product amplified using primers #71 and #67 inserted between EcoRI-BamHI sites in pLKB4
pAKW1073	*cyaT18-parB*$_{1-149}$; PCR product amplified using primers #66 and #73 inserted between EcoRI-BamHI sites in pLKB4
pAKW1072	*cyaT18-parB*$_{1-36}$; PCR product amplified using primers #66 and #72 inserted between EcoRI-BamHI sites in pLKB4
pAKW1098	*cyaT18-parB*$_{L8A}$; PCR fragments amplified with primers #74/ #8 and #7/ #19 on pAKW1075 were assembled in an overlap PCR (primers #74, #19) and inserted between EcoRI-BamHI sites in pLKB4. Eco52I site introduced through silent mutagenesis.
pAKW1099	*cyaT18-parB*$_{G9A}$; PCR fragments amplified with primers #74/ #10 and #9/ #19 on pAKW1075 were assembled in an overlap PCR (primers #74, #19) and inserted between EcoRI-BamHI sites in pLKB4. Eco72I site introduced through silent mutagenesis.
pAKW1100	*cyaT18-parB*$_{R10A}$; PCR fragments amplified with primers #74/ #12 and #7/ #11 on pAKW1075 were assembled in an overlap PCR (primers #74, #19) and inserted between EcoRI-BamHI sites in pLKB4. XbaI site introduced through silent mutagenesis.
pAKW1101	*cyaT18-parB*$_{G11A}$; PCR fragments amplified with primers #74/ #14 and #7/ #13 on pAKW1075 were assembled in an overlap PCR (primers #74, #19) and inserted between EcoRI-BamHI sites in pLKB4. BssHII site introduced through silent mutagenesis.
pAKW1102	*cyaT18-parB*$_{L12A}$; PCR fragments amplified with primers #74/ #16 and #7/ #115 on pAKW1075 were assembled in an overlap PCR (primers #74, #19) and inserted between EcoRI-BamHI sites in pLKB4. NsiI site introduced through silent mutagenesis.
pKGB0428	*cyaT18-ndpA* (*PA3849*), PCR product amplified using primers #75 and #76 inserted between EcoRI-BamHI sites in pLKB4
pAKW0798	*cyaT18-ndpA2*(*D3C65_RS24150*), PCR product amplified using primers #78 and #79 inserted between EcoRI-BamHI sites in pLKB4
pAAB0694	*cyaT18-PA4465*; PCR product amplified using primers #81 and #82 inserted between EcoRI-KpnI sites in pLKB4
pKGB0377	*cyaT18-aruC*(*PA0895*); PCR product amplified using primers #84 and #85 inserted between EcoRI-BamHI sites in pLKB4
pKGB0425	*cyaT18-PA3637*; PCR product amplified using primers #90 and #91 inserted between EcoRI-BamHI sites in pLKB4
pKGB0376	*cyaT18-PA4602*; PCR product amplified using primers #87 and #88 inserted between EcoRI-BamHI sites in pLKB4
pKGB0393	*cyaT18-PA4920*; PCR product amplified using primers #93 and #94 inserted between EcoRI-BamHI sites in pLKB4
pKGB0394	*cyaT18-PA5172*; PCR product amplified using primers #96 and #97 inserted between EcoRI-BamHI sites in pLKB4
pKGB0021	*cyaT18-minC*(*PA3243*); PCR product amplified using primers #101 and #115 inserted as an EcoRI-SalI fragment in pBGS18 to yield pKGB0015. *minC* excised as an EcoRI-HincII fragment and inserted between EcoRI-SmaI sites of pLKB4.
pKGB0003	*cyaT18-minD*(*PA3244*); PCR product amplified using primers #103 and #116 inserted as EcoRI-SalI fragment in pBGS18 to yield pKGB0001. Subsequently *minD was excised* as the EcoRI-Eco47III fragment and inserted between EcoRI-SmaI sites of pLKB4.
pKGB0004	*cyaT18-minE*(*PA3245*); PCR product amplified using primers #103 and #117 inserted as an EcoRI-SalI fragment in pBGS18 to yield pKGB0002. Subsequently *minE was excised* as an EcoRI-HincII fragment and inserted between EcoRI-SmaI sites of pLKB4.
pKGB0038	*cyaT18-PA5028*; PCR product amplified using primers #111 and #121 inserted between EcoRI-BamHI sites in pLKB4
pKGB0037	*cyaT18-fleN*(*PA1454*); PCR product amplified using primers #113 and #118 inserted between EcoRI-BamHI sites in pLKB4
pKGB0010	*cyaT18-PA3481*; PCR product amplified using primers #109 and #120 inserted between EcoRI-BamHI sites in pLKB4
pKGB0009	*cyaT18-PA1462*; PCR product amplified using primers #107 and #119 inserted between EcoRI-BamHI sites in pLKB4
pKGB0422	*cyaT18-PA4215*; PCR product amplified using primers #135 and #136 inserted between EcoRI-BamHI sites in pLKB4
pMKU0312	*cyaT18-PA3029*; PCR product amplified using primers #164 and #165 digested with EcoRI-BglII and inserted between EcoRI-BamHI sites in pLKB4
pKGB0400	*cyaT18-PA4217*; PCR product amplified using primers #137 and #138 inserted between EcoRI-BamHI sites in pLKB4
pAKW0524	*cyaT18-PA1801*; PCR product amplified using primers #139 and #140 inserted between EcoRI-BamHI sites in pLKB4
pKGB0426	*cyaT18-PA4673*; PCR product amplified using primers #141 and #142 inserted between EcoRI-BamHI sites in pLKB4
pKGB0427	*cyaT18-PA1804*; PCR product amplified using primers #144 and #145 inserted between EcoRI-BamHI sites in pLKB4
pKGB0438	*cyaT18-PA1609*; PCR product amplified using primers #146 and #147 inserted between EcoRI-BamHI sites in pLKB4
pAKW0525	*cyaT18-PA3326*; PCR product amplified using primers #149 and #150 inserted between EcoRI-BamHI sites in pLKB4
pAKW0137	*cyaT18-PA0964*; PCR product amplified using primers #151 and #152 digested with EcoRI-MunI and inserted into EcoRI site in pLKB4
pABB18.2	*cyaT18-smc*(*PA1527*); PCR product amplified using primers #191 and #192 inserted between EcoRI-BamHI sites in pLKB4
pKBB18.3	*cyaT18-scpA*(*PA3198*); PCR product amplified using primers #154 and #155 inserted between EcoRI-KpnI sites in pLKB4
pKBB18.5	*cyaT18-scpB*(*PA3197*); PCR product amplified using primers #157 and #158 inserted between EcoRI-KpnI sites in pLKB4
pMKU0310	*cyaT18-PA0205*; PCR product amplified using primers #160 and #161 digested with EcoRI-BglII and inserted between EcoRI-BamHI sites in pLKB4
pMKU0300	*cyaT18-PA0247*; PCR product amplified using primers #162 and #163 digested with EcoRI-BglII and inserted between EcoRI-BamHI sites in pLKB4
pMKU0292	*cyaT18-PA0411*; PCR product amplified using primers #168 and #169 digested with EcoRI-BglII and inserted between EcoRI-BamHI sites in pLKB4
pAKW0057	*cyaT18-PA0491*; PCR product amplified using primers #172 and #173 digested with EcoRI-MunI and inserted in EcoRI site in pLKB4
pMKU0306	*cyaT18-PA1466*; PCR product amplified using primers #174 and #175 digested with EcoRI-BglII and inserted between EcoRI-BamHI sites in pLKB4
pMKU0294	*cyaT18-PA2220*; PCR product amplified using primers #166 and #167 inserted between BamHI-SacI sites in pUT18C
pMKU0311	*cyaT18-PA2468*; PCR product amplified using primers #176 and #177 digested with EcoRI-BglII and inserted between EcoRI-BamHI sites in pLKB4
pAKW0059	*cyaT18-PA4290*; PCR product amplified using primers #178 and #179 inserted between BamHI-SacI sites in pUT18C
pMKU0315	*cyaT18-PA4581*; PCR product amplified using primers #180 and #181 inserted between EcoRI-BamHI sites in pLKB4
pAKW0064	*cyaT18-PA4906*; PCR product amplified using primers #182 and #183 inserted between BamHI-SacI sites in pUT18C
pMKU0297	*cyaT18-PA4914*; PCR product amplified using primers #184 and #185 digested with EcoRI-BglII and inserted between EcoRI-BamHI sites in pLKB4
pAKW0065	*cyaT18-PA5338*; PCR product amplified using primers #187 and #188 inserted between BamHI-SacI sites in pUT18C
pMKU0292	*cyaT18-PA1529*; PCR product amplified using primers #170 and #171 digested with SalI-BglII and inserted between SalI-BamHI sites in puT18C
pMKU0294	*cyaT18-PA2504*; PCR product amplified using primers #189 and #190 digested with EcoRI-BglII and inserted between EcoRI-BamHI sites in pLKB4
**BACTH vectors (pUT18 derivatives)**	
pKGB0437	*ndpA*(*PA3849*)-*cyaT18*, PCR product amplified using primers #75 and #77 inserted between EcoRI-BamHI sites in pKGB4
pAKW0800	*ndpA2*(*D3C65_RS24150*)-*cyaT18*, PCR product amplified using primers #75 and #77 inserted between EcoRI-BamHI sites in pKGB4
pAKW1556	*PA4465-cyaT18*, PCR product amplified using primers #81 and #83 digested with EcoRI-EcoRV and inserted between EcoRI-SmaI sites in pKGB4
pKGB0407	*aruC*(*PA0895*)-*cyaT18*; PCR product amplified using primers #84 and #86 inserted between EcoRI-BamHI sites in pKGB4
pAKW0214	*mexZ*(*PA2020*)-*cyaT18*, PCR product amplified using primers #99 and #100 inserted between EcoRI-BamHI sites in pKGB4
pKGB0401	*PA4920-cyaT18*, PCR product amplified using primers #93 and #95 inserted between EcoRI-BamHI sites in pKGB4
pKGB0417	*PA5172-cyaT18*, PCR product amplified using primers #96 and #98 inserted between EcoRI-BamHI sites in pKGB4
pKGB0065	*minC*(*PA3243*)-*cyaT18*; PCR product amplified using primers #101 and #102 inserted between EcoRI-BamHI sites in pKGB4
pKGB0069	*minD*(*PA3244*)-*cyaT18*; PCR product amplified using primers #103 and #104 inserted between EcoRI-BamHI sites in pKGB4
pKGB0070	*minE*(*PA3245*)-*cT18*; PCR product amplified using primers #105 and #106 inserted between EcoRI-BamHI sites in pKGB4
pKGB0081	*PA5028-cyaT18*; PCR product amplified using primers #111 and #112 inserted between EcoRI-BamHI sites in pKGB4
pKGB0090	*fleN*(*PA1454*)-*cT18*; PCR product amplified using primers #113 and #114 inserted between EcoRI-BamHI sites in pKGB4
pKGB0068	*PA3481-cyaT18*; PCR product amplified using primers #109 and #110 inserted between EcoRI-BamHI sites in pKGB4
pKGB0064	*PA1462-cyaT18*; PCR product amplified using primers #107 and #108 inserted between EcoRI-BamHI sites in pKGB4
pKGB0419	*PA4215-cyaT18*; PCR product amplified using primers #135 and #193 inserted between EcoRI-BamHI sites in pKGB4
pKGB0424	*PA4217-cyaT18*; PCR product amplified using primers #137 and #194 inserted between EcoRI-BamHI sites in pKGB4
pKGB0449	*PA4673-cyaT18*; PCR product amplified using primers #141 and #143 inserted between EcoRI-BamHI sites in pKGB4
pKGB0431	*PA1804-cyaT18*; PCR product amplified using primers #144 and #195 inserted between EcoRI-BamHI sites in pKGB4
pKGB0439	*PA1609-cyaT18*; PCR product amplified using primers #146 and #148 inserted between EcoRI-BamHI sites in pKGB4
pAKW0139	*PA0964-cyaT18*; PCR product amplified using primers #151 and #153 inserted between EcoRI-BamHI sites in pKGB4
pABB18.1	*smc*(*PA1527*)-*cT18*; PCR product amplified using primers #191 and #197 inserted between EcoRI-BamHI sites in pKGB4
pKBB18.4	*scpA*(*PA3198*)-*cT18*; PCR product amplified using primers #154 and #156 inserted between EcoRI-BamHI sites in pKGB4
pKBB18.6	*scpB*(*PA3197*)-*cT18*; PCR product amplified using primers #157 and #159 inserted between EcoRI-BamHI sites in pKGB4
pAKW0090	*PA0491-cyaT18*; PCR product amplified using primers #172 and #196 digested with EcoRI-MunI and inserted in EcoRI site in pKGB4
**BACTH vectors (pKT25 derivatives)**	
pKGB0434	*cyaT25-ndpA(PA3849)*; pKLB2 with EcoRI-BamHI fragment containing *ndpA* from pKGB0428
pAKW0823	*cyaT25-ndpA2 (D3C65_RS24150)*; pKLB2 with EcoRI-BamHI fragment containing *ndpA2* from pAKW798
pAAB0719	*cyaT25-PA4465*; pKLB2 with EcoRI-KpnI fragment containing PA4465 from pAAB0694
pKGB0380	*cyaT25-aruC*(*PA0895*); pKLB2 with EcoRI-BamHI fragment containing *aruC* from pKGB0377
pKGB0447	*cyaT25-PA3637*; pKLB2 with EcoRI-BamHI fragment containing *PA3637* from pKGB0425
pKGB0379	*cyaT25-PA4602*; pKLB2 with EcoRI-BamHI fragment containing *PA4602* from pKGB0376
pKGB0555	*cyaT25-PA4920* ; pKLB2 with EcoRI-BamHI fragment containing *PA4920* from pKGB0393
pKGB0395	*cyaT25-PA5172*; pKLB2 with EcoRI-BamHI fragment containing *PA5172* from pKGB0394
pKGB0033	*cyaT25-minC*(*PA3243*); pKLB2 with EcoRI-KpnI fragment containing *PA5172* from pKGB0021
pKGB0005	*cyaT25-minD*(*PA3244*); pKLB2 with EcoRI-KpnI fragment containing *PA5172* from pKGB0003
pKGB0006	*cyaT25-minE*(*PA3245*); pKLB2 with EcoRI-KpnI fragment containing *PA5172* from pKGB0004
pKGB0035	*cyaT25-PA5028*; pKLB2 with EcoRI-BamHI fragment containing *PA5028* from pKGB0038
pKGB0028	*cyaT25-fleN*(*PA1454*); pKLB2 with EcoRI-BamHI fragment containing *fleN* from pKGB0037
pKGB0029	*cyaT25-PA3481*; pKLB2 with EcoRI-BamHI fragment containing *PA3481* from pKGB0010
pKGB0036	*cyaT25-PA1462*; pKLB2 with EcoRI-BamHI fragment containing *PA1462* from pKGB0009
pKGB0448	*cyaT25-PA4673*; pKLB2 with EcoRI-BamHI fragment containing *PA4673* from pKGB0426
pKGB0446	*cyaT25-PA1804*; pKLB2 with EcoRI-BamHI fragment containing *PA1804* from pKGB0427
pKGB0442	*cyaT25-PA1609*; pKLB2 with EcoRI-BamHI fragment containing *PA1609* from pKGB0438
pABB25.1	*cyaT25-smc*(*PA1527*); pKLB2 with EcoRI-BamHI fragment containing *smc* from pABB18.2
pKBB25.3	*cyaT25-scpA*(*PA3198*); pKLB2 with EcoRI-KpnI fragment containing *scpA* from pKKB18.3
pAKW0078	*cyaT25-PA0491*; pKLB2 with EcoRI-BamHI fragment containing *PA0964* from pAKW0057
pAKW0089	*cyaT25-PA4914*; pKLB2 with EcoRI-SmaI fragment containing *PA4914* from pMKU0297
pAKW0068	*cyaT25-PA2504*; pKLB2 with EcoRI-SmaI fragment containing *PA2504* from pMKU0294
**BACTH vectors (pKNT25 derivatives)**	
pKGB0453	*ndpA*(*PA3849*)-*cyaT25*; pKGB5 with EcoRI-BamHI fragment containing *ndpA* from pKGB0437
pAKW824	*ndpA2*(*D3C65_RS24150*)-*cyaT25*; pKGB5 with EcoRI-BamHI fragment containing *ndpA2* from pAKW0800
pAKW1557	*PA4465-cyaT25*; PCR product amplified using #81 and #83 digested with EcoRI-EcoRV and inserted between EcoRI-SmaI sites in pKGB5
pKGB0409	*aruC*(*PA0895*)-*cyaT25*; pKGB5 with EcoRI-BamHI fragment containing *aruC* from pKGB0407
pAKW0322	*mexZ*(*PA2020*)-*cyaT25*; PCR product amplified using primers #99 and #100 inserted between EcoRI-BamHI sites in pKGB5
pKGB0452	*PA3637-cyaT25*; PCR product amplified using primers #90 and #92 inserted between EcoRI-BamHI sites in pKGB5
pKGB0404	*PA4602-cyaT25*; PCR product amplified using primers #87 and #89 inserted between EcoRI-BamHI sites in pKGB5
pKGB0405	*PA4920-cyaT25*; pKGB5 with EcoRI-BamHI fragment containing *PA4920* from pKGB0401
pKGB0418	PA5172-*cyaT25*; pKGB5 with EcoRI-BamHI fragment containing *PA5172* from pKGB0417
pKGB0072	*minC*(*PA3243*)-*cyaT25*; pKGB5 with EcoRI-BamHI fragment containing *minC* from pKGB0065
pKGB0078	*minD*(*PA3244*)-*cyaT25*; pKGB5 with EcoRI-BamHI fragment containing *minD* from pKGB0069
pKGB0079	*minE*(*PA3245*)-*cyaT25*; pKGB5 with EcoRI-BamHI fragment containing *minE* from pKGB0070
pKGB0094	*PA5028-cyaT25*; pKGB5 with EcoRI-BamHI fragment containing *PA5028* from pKGB0081
pKGB0093	*fleN*(*PA1454*)-*cyaT25*; pKGB5 with EcoRI-BamHI fragment containing *fleN* from pKGB0090
pKGB0077	*PA3481-cyaT25*; pKGB5 with EcoRI-BamHI fragment containing *PA3481* from pKGB0068
pKGB0092	*PA1462-cyaT25*; pKGB5 with EcoRI-BamHI fragment containing *PA1462* from pKGB0064
pKGB0420	*PA4215-cyaT25*; pKGB5 with EcoRI-BamHI fragment containing *PA4215* from pKGB0419
pKGB0421	*PA4217-cyaT25*; pKGB5 with EcoRI-BamHI fragment containing *PA4217* from pKGB0424
pKGB0450	*PA4673-cyaT25*; pKGB5 with EcoRI-BamHI fragment containing *PA4673* from pKGB0449
pKGB0451	*PA1804-cyaT25*; pKGB5 with EcoRI-BamHI fragment containing *PA1804* from pKGB0431
pKGB0444	*PA1609-cyaT25*; pKGB5 with EcoRI-BamHI fragment containing *PA1609* from pKGB0439
pABB25.2	*smc*(*PA1527*)-*cyaT25*-; pKGB5 with EcoRI-BamHI fragment containing *smc* from pABB18.1
pKBB25.4	*scpA*(*PA3198*)-*cyaT25*; pKGB5 with EcoRI-BamHI fragment containing *scpA* from pKBB18.4
pKBB25.6	*scpB*(*PA3197*)-*cyaT25*; pKGB5 with EcoRI-BamHI fragment containing *scpB* from pKBB18.6
pAKW0092	*PA0491-cyaT25*; pKGB5 with EcoRI-BamHI fragment containing *PA0491* from pAKW0090
**Expression vectors**	
pAKW1125	*tac*p-*yfp-parB*; *yfp* amplified using primers #122 and #123 on pEYFP and inserted in the EcoRI site in pABB1.2 yielding pASB1.2. Linker (GGAG) introduced between YFP and ParB by site directed mutagenesis of pASB1.2 using primers #124 and #125 yielding pABB0596. SacI in the vector removed through partial digestion of pABB0596, Klenow fill-in and self ligation.
pAKW1126	*tac*p-*yfp-parB*$_{L8A}$; PCR product amplified using primers #124 and #19 on pAKW1098 replaced SacI-NruI fragment in pAKW1125
pAKW1128	*tac*p-*yfp-parB*$_{R10A}$; PCR product amplified using primers #124 and #19 on pAKW1100 replaced SacI-NruI fragment in pAKW1125
pAKW1129	*tac*p-*yfp-parB*$_{G11A}$; PCR product amplified using primers #124 and #19 on pAKW1101 replaced SacI-NruI fragment in pAKW1125
pAKW1130	*tac*p-*yfp-parB*$_{L12A}$; PCR product amplified using primers #124 and #19 on pAKW1102 replaced SacI-NruI fragment in pAKW1125
pABB0543	*tac*p-*parA*(*PA5563*); EcoRI-KpnI fragment from pMKB5.1 containing *parA* inserted into pAMB9.37.
pKGB0218	*tac*p-*PA5028*; EcoRI-KpnI fragment from pKGB0010 containing *PA5028* inserted into pAMB9.37.
pKGB0223	*tac*p-*fleN*(*PA1454*); EcoRI-KpnI fragment from pKGB0037 containing *fleN* inserted into pAMB9.37.
pKGB0222	*tac*p-*PA3481*; EcoRI-KpnI fragment from pKGB0038 containing *PA3481* inserted into pAMB9.37.
pAKW1474	*tac*p-*PA4465*; EcoRI-KpnI fragment from pAAB0694 containing *PA4465* inserted into pAMB9.37.
pAKW1483	*tac*p-*ndpA*(*PA3849*); EcoRI-KpnI fragment from pKGB0428 containing *ndpA* inserted into pAMB9.37.
pAAB0595	*tac*p-*parA-cfp*; pECFP modified by EcoRI digestion and Klenow fill-in to yield pASB2. *parA* amplified using primers #131 and 132 and inserted into pASB2 as KpnI-NcoI fragment to yield pASB2.1. *parA-cfp* excised with EcoRI, SalI and inserted into pGBT30 to yield pASB3. *tac*p-*parA-cfp* amplified using primers #133 and #134 on pASB3 and inserted as an XbaI-SacI fragment into pAMB9.37.
pKGB0247	*tac*p-*yfp-PA3481*; *yfp* amplified using primers #122 and #123 on pEYFP and inserted in EcoRI site in pKGB0222
pKGB0245	*tac*p-*yfp-PA5028*; *yfp* amplified using primers #122 and #123 on pEYFP and inserted in EcoRI site in pKGB0218
pAKW1476	*tac*p-*sfGFP-PA4465*; *sfGFP* amplified from Bba_I746908 using primers #126 and #127 , digested with EcoRI and sub-cloned in pUC19 yielding pAKW1263. *sfGFP* excised as an EcoRI fragment and inserted into the EcoRI site of pAKW1474.
pAKW1298	*tac*p-*sfGFP-fleN*(*PA1454*); *sfGFP* excised as an EcoRI fragment from pAKW1263 and inserted into the EcoRI site of pKGB0223.
pAKW1284	*tac*p-*fleN*(*PA1454*)-*sfGFP*; *sfGFP* amplified from Bba_I746908 using primers #128, #129 and cloned as EcoRI-SalI in pUC19 yielding pAKW1199 (*sfGFP* preceded by EcoRI, EcoRV and BamHI sites). *fleN* amplified from pKGB0037 using primers #74 and #130 and cloned as an EcoRI-BamHI fragment in pAKW1199 to yield pAKW1220. EcoRI-KpnI fragment from pAKW1072 containing *parB*$_{1-36}$ inserted into pAMB9.37 yielding pAKW1156 (this introduced SmaI site into *mcs*). fleN-sfGFP transferred from pAKW1220 into pAKW1156 as EcoRI-SmaI fragment to yield pAKW1284.
pAKW1205	*araC-araBAD*p-*parB*$_{P1}$-*gfp*, *parB*$_{P1}$-*gfp* amplified using primers #64 and #65 on pOKB3, digested with EcoRI-SmaI and inserted between EcoRI-HincII sites of pKAB8
pKGB0067	*t7*p-*his*$_6$-*PA5028*; pET28a with EcoRI-SacI fragment from pKGB0038
pKGB0066	*t7*p-*his*$_6$-*fleN*(*PA1454*); pET28a with EcoRI-SacI fragment from pKGB0037
pKGB0063	*t7*p-*his*$_6$-*PA3481*; pET28a with EcoRI-SacI fragment from pKGB0010

Table S8 Oligonucleotides used in this study

Oligo nr	Common name	Sequence 5'-3'
#1	FLparB_F	TGACAAGCTTGCTGGAAATGGACATGAAGG
#2	DparB5_R	TGACCTGCAGGCGGGTTCCTTATGCGG
#3	DparB3_F	TGACCTGCAGCACACTTTGGGTTGTAGCG
#4	DparB3_R	TGACGTCGACGACCATCAGGGTCAGCAGAT
#5	gidB1	CCGAATTCATGTCTGCGGTAACCCA
#6	delparA1	GCAAGCTTCATGGTTATCCCTGTTCCCCC
#7	B_L8A_F	GTGGAGCCGGCCGCGGGCTCGATGC
#8	B_L8A_R	AGCCCGCGGCCGGCTCCACGTTTCTTGGCTGC
#9	B_G9A_F	ACTGGCACGTGGGCTCGATGCCCTGCT
#10	B_G9A_R	AGCCCACGTGCCAGTCCACGTTTCTTGGC
#11	B_R10A_F	GCCGGTCTAGATGCCCTGCTGGGC
#12	B_R10A_R	GCATCTAGACCGGCTCCCAGTCCACGTTTCTTG
#13	B_G11A_F	GGACGCGCGCTCGATGCCCTGCTGGG
#14	B_G11A_R	ATCGAGCGCGCGTCCCAGTCCACGTTTCTTG
#15	B_L12A_F	GGGCCGATGCATTGCTGGGCGGCTCG
#16	B_L12A_R	AGCAATGCATCGGCCCCGCGTCCCAGTCC
#17	FLB_OL_R	TCGTCGTCCTTGTAGTCCATGCGGGTTCCTTATGCGG
#18	FLB_OL_F	CCGCATAAGGAACCCGCATGGACTACAAGGACGACGA
#19	FLparB_R	TGACGTCGACCTTCCTCGATCGGGTTGA
#20	EsmcupF	GCGAATTCCTGGTCATCCAGCAGCGCCTG
#21	HsmcupR	CGAAGCTTCTTCAGGCGCATGCGTGCGCTCCC
#22	HsmcdwF	GGAAGCTTGCTGAAGCCTGAGTGTCAGGCAGTG
#23	SsmcdwR	CCGTCGACCAGCAGTTCAGCCGAGTCGCCGTC
#24	1 (CN1)	GGAATTCGCGACTTTTCCGGAAAGCAC
#25	2 (CN2)	GAAGCTTTACGGGGTCTTGATCGAGGA
#26	3 (CC1)	GAAGCTTCTGTCGGGTGACGTGTTGAA
#27	4 (CC2)	GGGATCCCCATGACTTTCTCGACGCCT
#28	5 (DN1)	GAAGCTTATCCATGTGTACGGCCCGAT
#29	6 (DN2)	GCCATGGTTGGCCAAGGTGATTCACCC
#30	7 (DC1)	GCCATGGACCGCATCGATTCCTCGATG
#31	8 (DC2)	GGGATCCTTCCACCTGGATCTGCTCCT
#32	11 (EC1)	GCCATGGACCCTGCCGGATCGTTGATT
#33	12 (EC2)	GGGATCCCGGTATAGGCTTTCTCGGTC
#34	fleNN1	GGAATTCGGGTTGCATCCTGACTAAGC
#35	fleNN2	GAAGCTTCTGTACGGGATGCATGCTAC
#36	fleNC1	GAAGCTTACAGGTTCGGCCGTATGACA
#37	fleNC2	GGGATCCTCGGCAGCAACTTCGTGATC
#38	5028N1	GGAATTCGAGGAGCCCTTCGTGTTCTT
#39	5028N2	GAAGCTTTCTGATTGAACACCACGCGC
#40	5028C1	GAAGCTTTCGAAAGCTGAGCAGCCTCT
#41	5028C2	GGGATCCTTTTCCCAGTGCGCCTGTAC
#42	4465UPEF	CGGAATTCATGATCCGACTTGAGCAAATCC
#43	4465UPHR	GCAAGCTTATCACATGTCATTGCCCGTTCTGTAC
#44	4465dwEF	GCAAGCTTAGCCCATAACAAGAAGGACAG
#45	4465DWBR	CGGGATCCGAGGCTACTCGCCTTCGTCG
#46	mksEco5F	TATAGAATTCCAGTTACCGGAAAGCAACC
#47	mksHin5R	TATAAAGCTTGATCATCGGCTCGACGTACT
#48	mksHin3F	TATAAAGCTTTACGACAACCTGGAACACCA
#49	mksSal3R	TATAGTCGACATCAGGTGCAGCAACAGGTA
#50	PA3849N1	GGGAATTCGCTGTATCGCTGGCGCATC
#51	PA3849N2	GGAAGCTTGATCGGCATGTCAGGTTCGA
#52	PA3849C1	GGAAGCTTCGCAAGGACTGACCCGCG
#53	PA3849C2	GGGGATCCCCTGCGCTGGACGGCGC
#54	NdpIde5F	TATAGAATTCGTCGCTGCCTTCAGGAAC
#55	NdpIde5R	TATAAAGCTTTCAGAAAGACTCCTGGTTCAGG
#56	NdpIde3F	TATAAAGCTTGCTCCAGGATCGAGTACGAC
#57	NdpIde3R	TATAGGATCCCGTAGCTGGCTTCGAACTCT
#58	82R_EcoF	TATAGAATTCCCGCGTACATGCTCCTG
#59	82R_SHPR	CCCGGGAAGCTTCTGCAGGTTTCCGGCTTCCCGG
#60	82R_PHSF	CTGCAGAAGCTTCCCGGGGTCGGAAGCAGCCGTC
#61	82R_BamR	TATAGGATCCCGCCGAGGTGACCAAG
#62	parSP1NF	TATACCCGGGATGCATTGTAAAACGACGGCCAGTG
#63	parSP1NR	TATACCCGGGATGCATCAGGAAACAGCTATGACCATGA
#64	parBP1F	GCGAATTCATGTCAAAGAAAAACAGACC
#65	GFPsmaR	CGCCCGGGTTATTTGTAGAGCTCATCC
#66	ParB1	CCGAATTCATGGCAGCCAAGAAACGTGG
#67	ParBC4BR	TATAGGATCCTCATCAACGGATGTGGGCGA
#68	ParBN1EF	TATAGAATTCATGCAGCAGATCCCCCTCGA
#69	ParBN2EF	TATAGAATTCATGCTGACCCAGCAGCAGGTC
#70	ParBN3EF	TATAGAATTCATGCCGGTCAAGAGCGACCC
#71	ParBN5EF	TATAGAATTCATGCGTGGACTGGGACGCG
#72	ParBC1BR	TATAGGATCCTCACAACTCGCGGCGGTC
#73	ParBC2BR	TATAGGATCCTCACTGGAACTCCTGCTGCAAG
#74	pUT18_F	CGGATGTACTGGAAACGGTG
#75	PA3849F	GGGAATTCATGCCGATCCATCACGCCAT
#76	PA3849R	GGGGATCCTCAGTCCTTGCGCCGCTTG
#77	PA3849NR	GGGGATCCCGTCCTTGCGCCGCTTGAGC
#78	NdpIEc_F	TATAGAATTCATGCCTATCAAACACGCCA
#79	NdpIBa_R	TATAGGATCCTCATGACGTGCTCTCCTGC
#80	NdpIPUTR	TATAGGATCCCTGACGTGCTCTCCTGCTCC
#81	4465EF	GCGAATTCATGCGCCTGATCATCGTCAGC
#82	4465HKR	GCGGTACCAAGCTTCTAGCTGCTGAGATCGCGG
#83	4465BS2R	TATAGATATCCTCGAGGCTGCTGAGATCGCGGT
#84	PA0895F	GGGAATTCATGTCCGCTCCCCATGCG
#85	PA0895R	GGGGATCCTCAGCCGCGGACCAGCTT
#86	PA0895RN	GGGGATCCCGCCGCGGACCAGCTTGG
#87	PA4602F	GGGAATTCATGTTCAGCCGTGATTTGACC
#88	PA4602R	GGGATCCTTAGTTGCCGTAGACCGGGAA
#89	PA4602RN	GGGGATCCCGTTGCCGTAGACCGGGAACT
#90	PA3637F	GGGAATTCATGACGCGCTACATCTTCGTC
#91	PA3637R	GGGGATCCTCAGGCCTTCCCGGAATATTT
#92	PA3637NR	GGGGATCCCGGCCTTCCCGGAATATTTCAG
#93	PA4920F	GGGAATTCATGCAACAGATCCAACGCGA
#94	PA4920R	GGGGATCCTCAGGGCGCCTTCGGCAG
#95	PA4920RN	GGGGATCCCGGGCGCCTTCGGCAGTTC
#96	PA5172F	GGGAATTCATGGCTTTCAACATGCACAAC
#97	PA5172R	GGGGTACCTCAGATGTCGGCGAGGGTC
#98	PA5172NR	GGGGTACCCGATGTCGGCGAGGGTCGA
#99	2020_E1F	TATAGAATTCATGGCCAGGAAAACCAAAG
#100	2020_BaR	GTATGGATCCCGGCGTCCGCCAGCAA
#101	minCup	CCGAATTCATGAGCCAAGCCGACCTCCTCGATC
#102	minCNd	GGATCCCAAGGCGGGTGATGTTCAACACGT
#103	minD1	GAATTCTTGGCCAAAATTCTCGTA
#104	minDNd	GGATCCCTTCACGTCCTCCGAACA
#105	minE1	GAATTCATGAGCCTTTTAGACTTC
#106	minENd	GGATCCCACGATCCGGCAGGGTGA
#107	pa1462A	CCGAATTCATGAAAGTCTGGGCAGTC
#108	pa1462Nd	GGATCCCGGCCACCCGGGTGGCCGGCT
#109	pa3481A	CCGAATTCATGTCCGCTATCACTCG
#110	pa3481Nd	GGATCCCGTCGTCGCTGATGCTGATGT
#111	pa5028A	CCGAATTCATGCGGCGCGTGGTGTTCAATCAG
#112	pa5028Nd	GGATCCCGCTTTCGAGCAGCCCGTGCAACTCGA
#113	FleNA	CCGAATTCATGAAGCAGATGGGTAGCATG
#114	fleNNd	GGATCCCTACGGCCGAACCTGTCGCGGGGT
#115	MinCdown	GCGTCGACCCTGAAAGTGGCGGCAGTATCCGT
#116	minD2	CTGCAGTCATTCACGTCCTCCGAA
#117	minE2	GTCGACTCAACGATCCGGCAGGGT
#118	pafleNb	CCGGATCCGAGCTCCGCCTTGCTATACATACG
#119	pa1464B	GCGGATCCGAGCTCGTCTACAACGGTTATCGG
#120	pa3481B	CCGGATCCGAGCTCTGATCGCGAATCTTACC
#121	pa5028B	CCGGATCCGAGCTCGTTACCTCGTAGAAGCGCTG
#122	YFPLeco	GCGAATTCATGGTGAGCAAGGGCGAGGA
#123	YFPPeco	GCGAATTCCTTGTACAGCTCGTCCATGC
#124	linkerB	GGACGAGCTCTACAAGGAAGGAGGTGCAGGTATGGCAGCCAAGAAACG
#125	LINKERbr	CCACGTTTCTTGGCTGCCATACCTGCACCTCCTTCCTTGTAGAGCTCG
#126	sfGFPEcF	TATAGAATTCATGCGTAAAGGCGAAGAGC
#127	sfGFPEMR	TATAGAATTCCAATTGACCGGAACCGGATCCTTTG
#128	sfGFPLiF	TATAGAATTCGATATCGGTTCCGGATCCGGTATG
#129	sfGFPSaR	TATAGTCGACCCGGGTCATTTGTACAGTTCATCCATACCA
#130	FleNBsBR	TATAGGATCCTACGGCCGAACCTGTCG
#131	KpnEcoAF	GCGGTACCGAATTCATGGCTAAGGTATTCGCGATCG
#132	NcoparAR	CGCCATGGCTGCGGTTGCGGCGCGGCC
#133	SacItacF	GCGAGCTCCCGTTCTGGATAATG
#134	XbacfpR	CGTCTAGATTCCTTACTTGTACAGCTCGTCCATG
#135	PA4215F	GGGAATTCATGCACAGATATGTCGTGATAG
#136	PA4215R	GGGGATCCTCACAGGTAGGCGCGGCC
#137	PA4217F	GGGAATTCATGAGCGAACCCATCGATATC
#138	PA4217R	GGGGATCCCTAGCGTGGCCGTTCCACC
#139	1801EcoF	TATAGAATTCATGTCTCGCAACTCTTTTATTCC
#140	1801BamR	TATAGGATCCTTAGACGGCCAGGTCGC
#141	PA4673F	GGCAATTGATGGGATTCAACTGCGGCATC
#142	PA4673R	GGGGATCCTTACACGTTGAAGCGGAAATGC
#143	PA4673NR	GGGGATCCCCACGTTGAAGCGGAAATGCAT
#144	PA1804F	GGGAATTCGTGAACAAGTCGGAACTGATC
#145	PA1804R	GGGGATCCTTAGTTGACAGCATCCTTCAGG
#146	PA1609F	GGGAATTCATGCGTCGCGTCGTTATCAC
#147	PA1609R	GGGGATCCTCAACCCTGCCAGCGCTTG
#148	PA1609NR	GGGGATCCCACCCTGCCAGCGCTTGAG
#149	clpP2a	CCCTGCAGGAATTCATGAAAACCGATGACAAGGACC
#150	3326BamR	TATAGGATCCTCACTGGCCAGGCAGC
#151	0964_EcF	TATAGAATTCATGGCTGGTCATTCTAAATGG
#152	0964_MfR	ATATCAATTGTCAGCCGAGCTGCGCCA
#153	0964_BSR	TATAGGATCCTGCCGAGCTGCGCCATCA
#154	scpAEcoF	GCGAATCATGTTGGAGGTCTTCCTCGAGG
#155	scpAKpnR	GCGGTACCGTCCGCAGGAGGACGAAC
#156	scpACTR	GCGGTACCCCTCGAAATCGTCTTCCAGG
#157	scpBEcoF	GCGAATTCATGAACCTGTCCGATCCCC
#158	scpBKpnR	GCGGTACCGGGTGGAACGACGGTCAG
#159	scpBCTR	GCGGTACCCGCGGCGCGGCCAGTCGTCG
#160	0205F	CGGAATTCATGCATCCGATCCCTTCCCC
#161	0205R	CGAGATCTCCCGGGCGTTATGGCGGCTGCCTTTC
#162	0247F	GCGAATTCATGAAGACTCAAGTCGCCA
#163	0247R	GCAGATCTCCCGGGCTACTCGATTTCCTCGTAG
#164	3029F	GCGAATCCATGAGCCACAAGGCCGAAAC
#165	3029R	GCAGATCTCCCGGGACGTATCGATAGGCGTTTGC
#166	2220F	CGGGATCCATGATCAAACGTAACCTC
#167	2220R	CGGAGCTCAAGCTTACTGATAGTTAATAACCC
#168	0411F	CGGAATTCATGAAGAAAATCAACGCAGG
#169	0411R	CGAGATCTCCCGGGATGACGGAAGAAACGGGTTT
#170	PA1529F	CGGTCGACCATGACCGACACCCAGGC
#171	PA1529R	CGAGATCTGCATGCATCTTTCGCAGCAGCGCCGC
#172	0491_EcF	TATAGAATTCATGCTCCCCATGGCCCT
#173	0491_MfR	ATATCAATTGTCAGCGCCGGCTGCT
#174	1466F	GCGAATTCATGGAACCTGTACTGTTTTATGGCG
#175	1466R	GCAGATCTGTCGACTGCACGAAGCCAAGGGCAAGT
#176	2468F	GCGAATTCATGTTCGCCCCGATCCTTCT
#177	2468R	GCAGATCTCCCGGGTCATTCGCCGTAGCGCAGCA
#178	P4290_BF	TATAGGATCCAATGCAGCCCGCTCGTT
#179	P4290_BR	ATATGAGCTCCTAGCCGTTCAAGGCCA
#180	4581_F	GCGAATTCATGAAACGCACGGTCGCCAT
#181	4581_R	GCGGATCCGTCGACTGGTGTTCAGGCGCGTATCT
#182	P4906_BF	TATAGGATCCATGAGCAAACCGGGCCA
#183	P4906_SR	ATATGAGCTCCTAGTCGGCGGCGACGT
#184	4914_EcF	TATAGAATTCATGCTGAACAAACGCCG
#185	4914R	CGAGATCTGTCGACAAACGTCAAACCGGCCCGT
#186	M4914MfR	ATATCAATTGGAACCGGGCCGTGGC
#187	P5338_BF	TATAGGATCCTTGCCGGGCATAGACG
#188	P5338_SR	ATATGAGCTCTCAGCTACGCAGGCGG
#189	2504_EcF	TATAGAATTCATGGCCTCGTGGACCTT
#190	2504R	CGAGATCTGTCGACAGGTGCCAGGCTGAGGCTCAAAGC
#191	ECOSMCF	CCGAATTCATGCGCCTGAAGAGCATCAAG
#192	smcRbis	GCGGATCCACTGCCTGACACTCAGG
#193	PA4215NR	GGGGATCCCCAGGTAGGCGCGGCCTTC
#194	PA4217NR	GGGGATCCCGCGTGGCCGTTCCACCTG
#195	PA1804NR	GGGGATCCCGTTGACAGCATCCTTCAGGG
#196	MO491MfR	ATATCAATTGGGCGCCGGCTGCTGCT
#197	SmcBamR	CCGGATCCCGGCTTCAGCCAATGCGACC

BIBLIOGRAPHY

1. El-Sayed AK, Hothersall J, Thomas CM. 2001. Quorum-sensing-dependent regulation of biosynthesis of the polyketide antibiotic mupirocin in *Pseudomonas fluorescens* NCIMB 10586. Microbiol Read Engl 147:2127–2139.
2. Ducret A, Quardokus EM, Brun YV. 2016. MicrobeJ, a tool for high throughput bacterial cell detection and quantitative analysis. 7. Nat Microbiol 1:1–7.
3. Medema MH, Takano E, Breitling R. 2013. Detecting sequence homology at the gene cluster level with MultiGeneBlast. Mol Biol Evol 30:1218–1223.
4. Katoh K, Frith MC. 2012. Adding unaligned sequences into an existing alignment using MAFFT and LAST. Bioinformatics 28:3144–3146.
5. Jalal AS, Tran NT, Stevenson CE, Chimthanawala A, Badrinarayanan A, Lawson DM, Le TB. 2021. A CTP-dependent gating mechanism enables ParB spreading on DNA. eLife 10:e69676.
6. Soh Y-M, Davidson IF, Zamuner S, Basquin J, Bock FP, Taschner M, Veening J-W, Rios PDL, Peters J-M, Gruber S. 2019. Self-organization of *parS* centromeres by the ParB CTP hydrolase. Science eaay3965.
7. Leonard TA, Butler PJG, Löwe J. 2004. Structural analysis of the chromosome segregation protein Spo0J from *Thermus thermophilus*. Mol Microbiol 53:419–432.
8. Chen B-W, Lin M-H, Chu C-H, Hsu C-E, Sun Y-J. 2015. Insights into ParB spreading from the complex structure of Spo0J and *parS*. Proc Natl Acad Sci 112:6613–6618.
9. Khare D, Ziegelin G, Lanka E, Heinemann U. 2004. Sequence-specific DNA binding determined by contacts outside the helix-turn-helix motif of the ParB homolog KorB. Nat Struct Mol Biol 11:656–663.
10. Kawalek A, Kotecka K, Modrzejewska M, Gawor J, Jagura-Burdzy G, Bartosik AA. 2020. Genome sequence of *Pseudomonas aeruginosa* PAO1161, a PAO1 derivative with the ICE*Pae*1161 integrative and conjugative element. BMC Genomics 21(1):1-12.
11. Hanahan D. 1983. Studies on transformation of *Escherichia coli* with plasmids. J Mol Biol 166:557–580.
12. Simon R, Priefer U, Pühler A. 1983. A broad host range mobilization system for *in vivo* genetic engineering: transposon mutagenesis in gram negative bacteria. Nat Biotechnol 1:784–791.
13. Karimova G, Pidoux J, Ullmann A, Ladant D. 1998. A bacterial two-hybrid system based on a reconstituted signal transduction pathway. Proc Natl Acad Sci 95:5752–5756.
14. Bartosik AA, Glabski K, Jecz P, Lasocki K, Mikosa M, Plochocka D, Thomas CM, Jagura-Burdzy G. 2014. Dissection of the region of *Pseudomonas aeruginosa* ParA that is important for dimerization and interactions with its partner ParB. Microbiology 160:2406–2420.
15. Jecz P, Bartosik AA, Glabski K, Jagura-Burdzy G. 2015. A Single *parS* sequence from the cluster of four sites closest to *oriC* is necessary and sufficient for proper chromosome segregation in *Pseudomonas aeruginosa*. PLoS One 10:e0120867.
16. Lasocki K, Bartosik AA, Mierzejewska J, Thomas CM, Jagura-Burdzy G. 2007. Deletion of the *parA* (*soj*) homologue in *Pseudomonas aeruginosa* causes ParB instability and affects growth rate, chromosome segregation, and motility. J Bacteriol 189:5762–5772.
17. Kawalek A, Glabski K, Bartosik AA, Fogtman A, Jagura-Burdzy G. 2017. Increased ParB level affects expression of stress response, adaptation and virulence operons and potentiates repression of promoters adjacent to the high affinity binding sites *parS3* and *parS4* in *Pseudomonas aeruginosa*. PLoS One 12:e0181726.
18. Reece KS, Phillips GJ. 1995. New plasmids carrying antibiotic-resistance cassettes. Gene 165:141–142.
19. Norrander J, Kempe T, Messing J. 1983. Construction of improved M13 vectors using oligodeoxynucleotide-directed mutagenesis. Gene 26:101–106.
20. Spratt BG, Hedge PJ, te Heesen S, Edelman A, Broome-Smith JK. 1986. Kanamycin-resistant vectors that are analogues of plasmids pUC8, pUC9, pEMBL8 and pEMBL9. Gene 41:337–342.
21. Jagura-Burdzy G, Ibbotson JP, Thomas CM. 1991. The korF region of broad-host-range plasmid RK2 encodes two polypeptides with transcriptional repressor activity. J Bacteriol 173:826–833.
22. Funnell BE. 1991. The P1 plasmid partition complex at *parS*. The influence of *Escherichia coli* integration host factor and of substrate topology. J Biol Chem 266:14328–14337.
23. Funnell BE. 1988. Mini-P1 plasmid partitioning: excess ParB protein destabilizes plasmids containing the centromere *parS*. J Bacteriol 170:954–960.
24. Kawalek A, Modrzejewska M, Zieniuk B, Bartosik AA, Jagura-Burdzy G. 2019. Interaction of ArmZ with the DNA-binding domain of MexZ induces expression of *mexXY* multidrug efflux pump genes and antimicrobial resistance in *Pseudomonas aeruginosa.* Antimicrob Agents Chemother 63(12), e01199-19.
25. Kovach ME, Phillips RW, Elzer PH, Roop RM, Peterson KM. 1994. pBBR1MCS: a broad-host-range cloning vector. BioTechniques 16:800–802.
26. Ludwiczak M, Dolowy P, Markowska A, Szarlak J, Kulinska A, Jagura-Burdzy G. 2013. Global transcriptional regulator KorC coordinates expression of three backbone modules of the broad-host-range RA3 plasmid from IncU incompatibility group. Plasmid 70:131–145.
27. Mierzejewska J, Bartosik AA, Macioszek M, Płochocka D, Thomas CM, Jagura-Burdzy G. 2012. Identification of C-terminal hydrophobic residues important for dimerization and all known functions of ParB of *Pseudomonas aeruginosa*. Microbiol Read Engl 158:1183–1195.
